# Highly ordered laser imprinted plasmonic metasurfaces for polarization sensitive perfect absorption

**DOI:** 10.1038/s41598-022-21647-w

**Published:** 2022-11-17

**Authors:** Anna C. Tasolamprou, Evangelos Skoulas, George Perrakis, Matina Vlahou, Zacharias Viskadourakis, Eleftherios N. Economou, Maria Kafesaki, George Kenanakis, Emmanuel Stratakis

**Affiliations:** 1 Institute of Electronic Structure and Laser, Foundation for Research and Technology Hellas, N. Plastira 100, Crete, 71110 Heraklion, Greece; 2 Department of Materials Science and Technology, University of Crete, 70013 Heraklion, Greece; 3 Department of Physics, University of Crete, 70013 Heraklion, Greece

**Keywords:** Metamaterials, Nanophotonics and plasmonics, Laser material processing, Nanowires

## Abstract

We present polarization-sensitive gap surface plasmon metasurfaces fabricated with direct material processing using pulsed laser light, an alternative and versatile approach. In particular we imprint laser induced periodic surface structures on nanometer-thick Ni films, which are back-plated by a grounded dielectric layer with TiO_2_ and ZnO deposition followed by Au evaporation. The procedure results in a metal-insulator-metal type plasmonic metasurface with a corrugated top layer consisting of highly-ordered, sinusoidal shaped, periodic, thin, metallic nanowires. The metasurface sustains sharp, resonant gap surface plasmons and provides various opportunities for polarization control in reflection, which is here switched by the size and infiltrating material of the insulating cavity. The polarization control is associated with the polarization sensitive perfect absorption and leads to high extinction ratios in the near-IR and mid-IR spectral areas. Corresponding Fourier-transform infrared spectroscopy measurements experimentally demonstrate that the fabrication approach produces metasurfaces with very well-defined, controllable, sharp resonances and polarization sensitive resonant absorption response which, depending on the insulating cavity size, impacts either the normal or the parallel to the nanowires polarization.

## Introduction

Metasurfaces are the two-dimensional counterpart of metamaterials, i.e., ultrathin electromagnetic periodic structures with an engineered response that depends on the architecture of the subwavelength elementary units, the meta-atoms^1^. Due to their versatile nature and the advanced electromagnetic control they offer, metasurfaces have been employed for a variety of applications, from shielding and steering to solar cell energy harvesting, sensing and more recently space-time modulators^2–8^, covering the vast majority of the electromagnetic spectrum, from microwave to THz and the optical frequencies^9–13^. Particularly interesting is the family of plasmonic metasurfaces operating in high frequencies where metals sustain Surface Plasmon Polaritons (SPPs), i.e., states propagating along, and tightly bound to, metal-dielectric interfaces^14^, while exhibiting relatively low dissipation losses and resonance-enabled potential for light manipulation, i.e., control over the amplitude, phase, direction and polarization of their impinging wave^15^. Closely related are the the so-called Gap Surface Plasmon Metasurfaces (GSPMs) that consist of a thin dielectric spacer sandwiched between two metallic uniform and/or periodically corrugated thin films, i.e., the also known metal-insulator-metal scheme. Due to the small thickness of the dielectric spacer (gap), the SPPs sustained in each individual metallic thin film undergo near-field coupling forming Gap Surface Plasmons (GSPs), the manipulation of which has led to a plethora of electromagnetic functionalities, including resonant perfect absorption^16–21^, which we study here.

The configuration of the metal-insulator-periodically corrugated metal metasurface is the most acclaimed scheme of implementing optical metasurfaces; it is a planar, relatively simple approach with the meta-atoms electromagnetic features further enhanced by the presence the uniform, reflecting metallic layer and the dielectric cavity^17,22^. The fabrication of metallic patterns on the micro or nano scale has been achieved with variable methods. Traditional methods usually include photolithography, particle beam lithography, direct-write lithography, pattern transfer, hybrid patterning lithography, and commonly involves noble metals^23^. In their majority these conventional techniques involve multiple step approaches, require controlled ambient conditions, such as vacuum-reactive gas environment and the use of toxic chemicals^24^. Here, we demonstrate that an alternative, environmentally friendly and versatile approach based on direct ultrafast laser processing of thin non-noble metallic films can be used for the growth of gap plasmon metasurfaces with well-defined and controllable resonant response. This approach is based on the selective generation of highly-ordered Laser Induced Periodic Surface Structures (LIPSS) implemented on nanometre-thick films^12,25,26^. LIPSS imprinting is inherently a single-step and large scale approach, metallic nanowire arrays are self-assembled onto a substrate with the use of laser pulses that induce and interfere with surface plasmon polaritons, in just a few minutes of time, and ambient environment^27–30^. It has been used in a variety of applications such as surface enhanced Raman scattering, enhanced thermal radiation emission efficiency, cell growth, wetting and more^31^. Metasurfaces related implementations that appear in literature, so far, mostly involve irregular LIPSS and/or imprinting in bulky metallic or dielectric substrates and/or imprinting in thin films without the inclusion of a plasmonic cavity as we present here, which results in relatively broad electromagnetic responses^12,32–35^. In our work, we make use of two key aspects necessary to enable high quality polarization dependent sharp responses: (i) the highly-ordered LIPSS that produce well-structured surface periodic corrugations and (ii) the thin film LIPSS implementation which allows the participation of the back-reflector and the formation of the insulating cavity to induce the gap surface plasmons resonances.

We focus on shaping resonant gap plasmonic states that provide polarization-dependent enhanced or perfect (near unity) absorption. Following the imprinting of the LIPSS on a 120 nm Ni thin film, we create a dielectric cavity upon TiO_2_ and ZnO deposition followed by gold (Au) evaporation which results in the metal-insulator-periodically corrugated metal type metasurface as shown in Fig. 3a. Two samples of different insulating spacer thickness are realized, one thinner with TiO_2_ cavity sample absorbing the polarization normal to the nanowires array, exhibiting a fundamental resonance at $\lambda \approx 1.5~\upmu$m, and another thicker sample with ZnO cavity absorbing the polarization parallel to the nanowires, exhibiting a higher order resonance at $\lambda \approx 2.5~\upmu$m. Fourier-Transform Infrared (FTIR) spectroscopy measurements demonstrate that the LIPSS imprinted gap plasmons metasurfaces present well-defined, theoretically predicted and experimentally verified resonances that provide efficient polarization control in the near-IR and mid-IR spectra. The paper is organized as follows: Initially we present the methods used for the sample preparation, then, we discuss the physical origins of the polarization dependent perfect absorption and finally, we present the sample characterization.

## Sample preparation

### LIPSS formation in the thin metallic film

**Figure 1:**
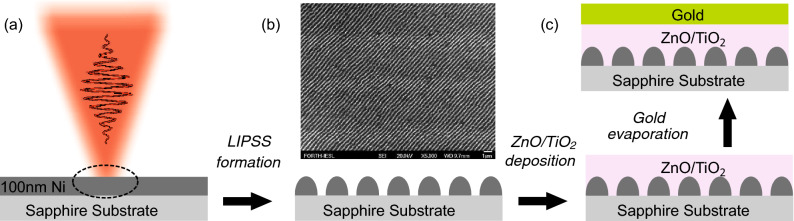
Schematic of the fabrication process employed for the realization of the gap plasmon metasurfaces. (**a**) LIPSS formation on a Ni thin film deposited on sapphire via irradiation with linearly polarized ultrashort laser pulses. (**b**) Schematic and top view SEM image of the LIPSS formed. (**c**) ZnO and TiO_2_ deposition followed by gold evaporation which results in the metal-insulator-periodically corrugated metal type metasurface.

**Figure 2:**
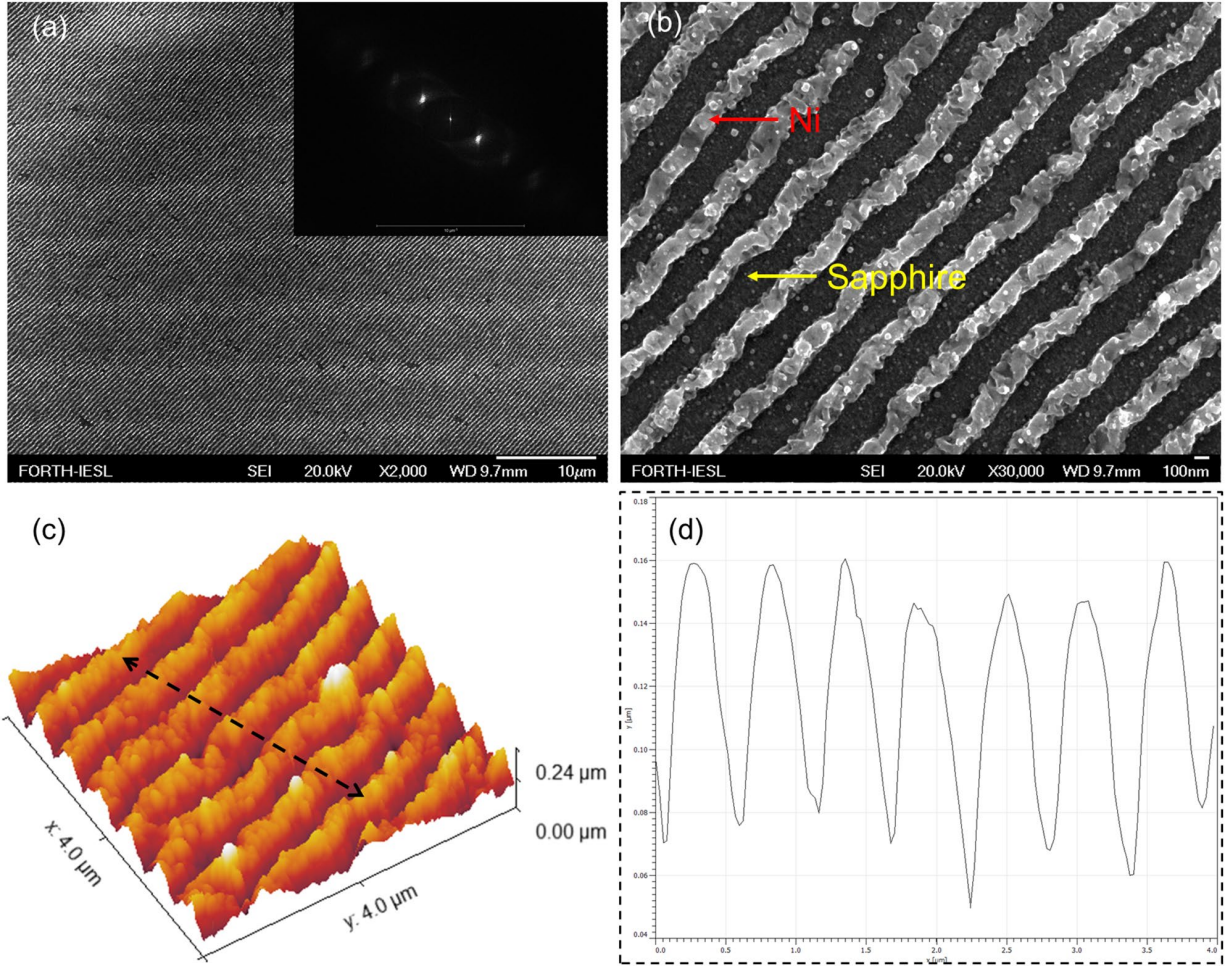
(**a**) Top view SEM image of the LIPSS formed on the thin Ni film, and (**b**) higher magnification. Inset in (**a**) shows the corresponding 2D-FFT. (**c**) Representative AFM image ($4\times 4$ μm) of LIPSS; (**d**) LIPSS cross-section along the double-ended arrow shown in (**c**).

**Figure 3:**
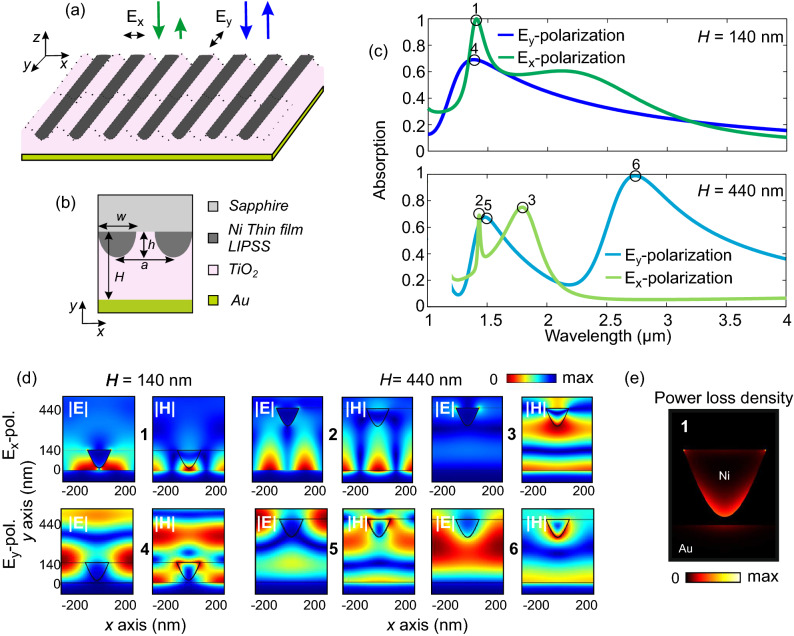
(**a**) Schematic of the LIPSS imprinted plasmonic gap metasurfaces and principle of operation for the polarizer, i.e., polarization sensitive perfect absorption, green arrows present the *x* polarization and blue arrows the *y* polarization. (**b**) Dimensions definition in the LIPSS imprinted metasurface. (**c**) Absorption spectra under normal incidence for two LIPSS imprinted, metal-insulator-metal, plasmonic gap metasurfaces of different insulating cavity thickness, one thinner with thickness $H=140$ nm (top panel) and one thicker with thickness $H=440$ nm (bottom panel) in the wavelength range $\lambda =$  [1–4] μm for both polarizations, i.e., the electric field being normal to the top layer nanowires, **E** = $E_x$ (dark- and light-green) and the electric field being parallel to the top layer nanowires **E** = $E_y$ (dark- and light-blue). (**d**) Distribution of the scattered absolute values of the electric and magnetic field in the selected wavelength points of the spectral response, points 1 to 6, for both polarizations shown in (**c**). Notice that the *y*-coordinate starts at $y=0$, at the interface between the gold layer and the dielectric layer and increases towards the Ni nanowire. (**e**) Distribution of the power loss density showing the plasmonic losses predominately in the Ni nanowire and then in the Au back-reflector.

The fabrication procedure involves the formation of the LIPSS, the deposition of the dielectric layer and the evaporation of gold, and it is shown schematically in Fig. 1. The first step is the imprinting of the LIPSS in the metallic thin film. Imprinting takes place in sub-ablation conditions, i.e., without the limitations of the laser beam focusing diffraction limit and the laser beam pointing stability, that would degrade the structures’ morphology which is important for assessing the metasurface resonant response. As a result of this process, which we in more detail discuss in Ref.^12^, extended areas of nanowires are attained via proper scanning of the laser beam onto the film surface with the periodicity, shape and size of the LIPSS defined by polarization control and a proper combination of irradiation conditions^27,28,34,36–38^. The experimental apparatus developed and used for the direct laser processing consists of an Yb:KGW Pharos-SP solid state laser (Light Conversion), emitting at $\lambda$ = 513 nm central wavelength, the repetition rate of which was set to 60 kHz and the pulse width of 170 fs. The samples were Ni thin metal films of 100 nm thickness, deposited with magnetron sputtering on an additional 5 nm Chromium coating. The substrates were 1 mm thick amorphous sapphire (Al_2_O_3_), supplied from Crystran. The samples were placed on a motorized servo 3-axis stage and all irradiations were performed in ambient conditions at room temperature. The laser beam was guided and focused on the sample surface with a 200 mm focal distance plano convex lens and all irradiations were performed at normal incidence, within the Rayleigh range of the focal position. The Gaussian spot size diameter, d(1/e^2^) $\sim$ 27 μm, was characterized by a CCD camera on the focal plane. The laser power was modulated from the laser amplifier settings and controlled with a half wave plate placed before a Glan Taylor polarizer. The typical dimensions of the formed LIPSS have been characterized by AFM analysis showing for the LIPSS feature^12^: an average height of $h=123\pm 20$ nm, an average width of $w=182\pm 66$ nm and an average periodicity of $a=462\pm 58$ nm, the definition of the dimensions in schematically shown in Fig. 3b. In Fig. 2 we provide a top view SEM image of the LIPSS formation on the thin film with lower (Fig. 2a) and higher (Fig. 2b) magnification. The inset in Fig. 2a shows the corresponding 2D-FFT while in Fig. 2c an AFM image of 4 × 4 μm area. The black dashed double ended arrow of Fig. 2c is presented at Fig. 2d as a cross-section of the ripple height. The increment on the LIPSS height is attributed to materials deposition during the laser processing. We considered and simulated the shape of the wire as sinusoidal or half-cylinder like as deducted for the surface analysis currently presented in Fig. 2. The half-cylinder like structure of the LIPSS is a result of the constructive interference that takes place at the interface between the air and thin Ni film. In particular, laser irradiation results in the formation of a surface plasmon polariton wave with the characteristic distribution intensity profile over the vertical direction that decays in the metal film at a distance defined by the skin depth of the material. The formation of the LIPSS follows the intensity distribution of the plasmon polariton and it is the reason why more of the thin film material is found on the bottom, near the sapphire substrate, and less on the top of the ripple. Taking into account the characteristics of the LIPSS, our tuning geometrical parameter, arranged in the next step of the fabrication, is the distance between the LIPSS and the Au back-reflector, i.e., the insulating cavity thickness.

### TiO_2_-ZnO deposition and Au evaporation

Following LIPSS formation, the samples were coated with either TiO_2_ or ZnO films, via spray pyrolysis and sol-gel/spin coating approaches, respectively. According to the theoretical analysis presented in detail in the next section, we fabricated two samples, a thinner one with a TiO_2_ cavity of 165 nm thickness which serves as a narrowband polarizer, and a thicker one with a ZnO cavity of 520 nm thickness which serves as a broader polarizer. We have selected two different insulating material following two different chemical techniques; TiO_2_ via sol-gel/spin-coating, ZnO via spray pyrolysis. This way we can verify the effectiveness of two fabrication approaches (for small and large scale samples, respectively), while the different refractive index of TiO_2_ and ZnO will affect the response of the metasurface as it is discussed in the next section. Regarding the TiO_2_ sample, a thin film with a thickness of 165 ± 10 nm was deposited on LIPSS structures following a sol-gel/spin coating approach, according to the methodology of Rampaul et al.^39^ and Kenanakis et al.^21,40^, its anatase TiO_2_ crystal structure has been veryfied by X-ray diffraction (XRD). Regarding the ZnO sample, a thin film was developed employing conventional spray pyrolysis technique, similar to the approaches of Yoshino et. al^41^ and Saha et. al^42^. In particular, a 0.2 M aqueous solution of zinc nitrate hexahydrate (Zn(NO_3_)_2_·6H_2_O, obtained from Sigma-Aldrich) was prepared and subsequently introduced in a commercially available air brush (nozzle diameter 0.3 mm). The latter was supplied with compressed air (pressure $\sim$ 1 bar), so as the aerosol was delivered to the preheated LIPSS substrate (110 °C) which has been positioned at 10 cm away from the air-brush nozzle. The whole process was performed in a fume hood, resulting in a ZnO coating of  520 ± 15 nm thickness. Then, the sample was left to cool down to room temperature. Its crystal structure was verified by X-ray diffraction (XRD) using a Rigaku (RINT 2000) diffractometer with Cu Ka X-rays revealing all the diffraction peaks are indexed to the ZnO hexagonal wurtzite structure^43^. Finally, a $\sim$200 nm thickness gold film was evaporated on the ZnO/TiO_2_ layers using a commercial thermal evaporator, with a deposition rate of about 0.5 Å/s.

## Polarization sensitive resonant absorption

The LIPSS imprinting process results in the formation of a metallic grid of sinusoidal like shaped wires with height $\sim$120 nm as seen in Fig. 3b. This sinusoidal cross-section is significantly different that the common gap plasmons metasurfaces that mainly consist of planar geometries like rectangular wires or patches with smaller thickness height, $\sim$30 nm^44^. However, as we show here, the structure is capable of providing very well-defined and sharp modes with polarization dependent enhanced or perfect (near unity) absorption. The resonant response is a function of all the participating material and geometrical parameters, the periodicity, the size and shape of the wire as well as on the thickness of the insulating cavity (i.e., the spacer between the Ni wire and the Au reflector). The most accessible degree of freedom for the design in the second step of the fabrication (ZnO and TiO_2_ deposition followed by gold evaporation) is the thickness of the insulating cavity in the metal-insulator-periodically corrugated metal metasurface, variable *H*, which we study here.

In such metasurfaces, depending on the thickness of the spacer, the nature of the resonance may undergo different transitions; when the spacer is too small the near-field effects dominate the cavity, and the resonance is accompanied by the excitation of a strong magnetic dipole, while, when the spacer gets larger the near-field effects become weaker and a transition from the clear magnetic dipole type resonance to a vertical Fabry-Perot resonance takes place^17^. To highlight these features for the case of the LIPSS imprinted, metal-insulator-periodically corrugated metal, plasmonic gap metasurfaces, we initially perform a theoretical analysis of the scattering spectra dependence on the cavity thickness, *H*. We use the commercial three-dimensional full-wave solvers CST Microwave Studio (Computer Simulation Technology GmbH, Darmstadt, Germany) and COMSOL Multiphysis. For the geometrical parameters we assume $h=120$ nm, $w=180$ nm and periodicity of 500 nm (see Fig. 3b for the dimensions definition) and regarding the material properties, the refractive index of the dielectrics involved is retrieved from Ref.^45–47^, while for Ni and Au we use the data from Ref.^48^. We solve for the scattering parameters, i.e., reflection and absorption (no transmission is possible behind the Au layer as the layer is much thicker than the skin depth), assuming the unit cell and periodic lateral conditions; see Fig. 3d. In Fig. 3c we present the absorption spectra under normal incidence for two of different TiO_2_ cavity thickness, one thinner with thickness $H=140$ nm (top panel) and one thicker with thickness $H=440$ nm (bottom panel), in the wavelength range $\lambda =$ [1–4] μm for both polarizations, i.e., the electric field being normal to the top layer nanowires, **E** = $E_x$ (dark- and light-green) and the electric field being parallel to the top layer nanowires **E** = $E_y$ (dark- and light-blue), see also Fig. 3a for the definition of the polarization. Figure 3d presents the distribution of the scattered electric and magnetic field (absolute values) for the thinner and thicker sample, for both polarizations, in selected wavelength points, see the circular markers 1 to 6 in Fig. 3c. Starting from the normal to the nanowires polarization, **E** = $E_x$, and the thinner sample (dark-green curve), in the top panel of Fig. 3c we can see that the structure presents a perfect absorption peak at around $\lambda =$1410 nm, point 1 in the dark-green curve, at a wavelength where the parallel polarization, **E** = $E_y$, is by 60% absorbed and by 40% reflected (see dark-blue curve). The distribution of the corresponding scattered field is shown in panels 1 of Fig. 3d. We can see that for these modes the near-field effects dominate while the resonance is characterized by the excitation of the fundamental magnetic dipole with the characteristic field distribution of the magnetic field^17^. The response is modified as the spacer thickness increases. In the bottom panel of Fig. 3c we present the absorption spectra for the thicker sample, with $H=440$ nm. As observed, for the **E** = $E_x$ polarization (light-green curve), a vertical Fabry-Perot, higher order resonance with degraded absorption peak appears at point 3, wavelength $\lambda$ = 1800 nm. A signature of the fundamental magnetic dipole mode survives, retaining the characteristic magnetic field distribution and appears as a sharp Fano-type resonance, as seen at point 2 of Fig. 3d, $\lambda$ = 1410 nm, of the $H=440$ nm case, light-green curve. Given the sharpness of the specific resonance, with proper adjustments the structure can be also effective for sensing applications^7^.

We should note here that the main contribution to the absorption mechanism comes from the plasmonic losses in the two metals. On the one hand we have the noble Au back-reflector and on the other hand, the refractory metal Ni. The incoming field is mostly dissipated in the sinusoidal Ni nanowire. This is due to the facts that (i) Ni exhibits a larger skin depth in the frequencies under consideration, $d_{Ni} \sim 35$ nm and $d_{Au} \sim 23$ nm, and (ii) the extended Ni surface that interacts with the exited mode with respect to Au. This is clearly shown in Fig. 3e where we present in the power loss distribution at the magnetic resonance (point 1 of Fig. 3c). As observed the major part of the losses occurs in the Ni wire. Interestingly non-noble metals like Ni that have higher reserve in nature than the high-cost noble metals, possess plasmonic behavior and intrinsic material absorption losses in a wide frequency and thus are gaining increasing attention for broadband, strong absorbing applications such as solar cell technology^49–51^.

The electromagnetic response of the parallel polarization, **E** = $E_y$, for the thinner and the thicker samples, is presented with the dark- and light-blue curves in the top and bottom panels of Fig. 3c, respectively. As observed, the electromagnetic response of the parallel polarization is less rich in features. The **E** = $E_y$ resonances have Fabry-Perot origins and this is evident in point 6 of the case $H=440$ nm, bottom panel, light-green curve in Fig. 3c and in the distribution presented in point 6 of Fig. 3d, where perfect absorption is achieved. The parallel polarization exhibits a perfect absorption peak at $\lambda$ = 2750 nm, with significantly larger bandwidth compared to the normal polarization in the thinner sample case, while the reflected power of the normal polarization at this wavelength is as high as 94%, see bottom panel light-green curves. Additionally for the **E** = $E_y$ we observe two resonances in the thicker sample (points 5 and 6 in Fig. 3c,d), and a single weaker resonance in the thin sample (point 4 in Fig. 3c,d).

It is evident that the difference in the response between the two polarizations is connected with the anisotropic shape of the metasurface. The impinging wave with a polarization normal to the nanowires, **E** = $E_x$, couples to the localized SPPs induced in the finite (along *x*) wires and effectively generates a resonant surface local current in each nanowire (with a direction orthogonal to the wire axis), followed by a opposite travelling current in the back-reflector. This is the physical origin of the induced magnetic mode observed in the normal polarization. On the other hand, in the parallel polarization, **E** = $E_y$, the incoming waves sense the nanowires essentially as a non-resonant uniform metallic sheet which leads to the formation of the Fabry-Perot resonances between the nanowires and the back-reflector.

**Figure 4:**
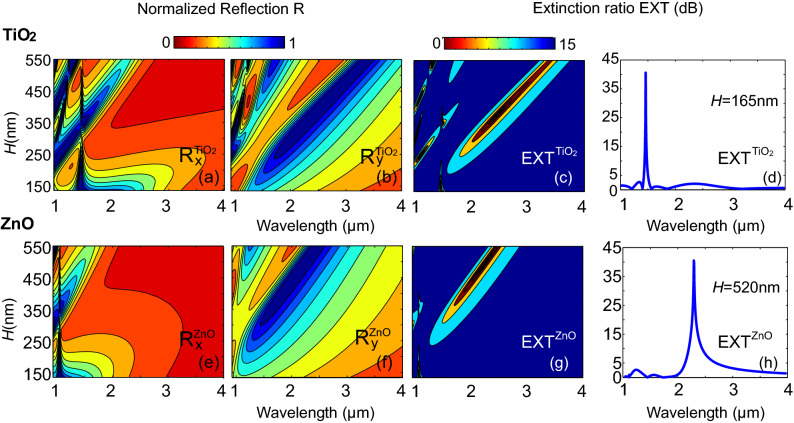
(**a**,**b**,**e**,**f**) Normalized reflected power, *R*, for two plasmonic gap metasurfaces of variable TiO_2_ [top panels (**a**) and (**b**)] and ZnO [bottom panels (**e**) and (**f**)] insulating cavity thickness in the range $H=135{-}550$ nm for both polarizations, i.e, the electric field being normal to the top layer nanowires, **E** = $E_x$ [panels (**a**) and (**e**)] and the electric field being parallel to the top layer nanowires **E** = $E_y$, [panels (**b**) and (**f**)]; R$_x^\mathrm {TiO_{2}}$ in (**a**), R$_y^\mathrm {TiO_{2}}$ in (**b**), R$_x^\mathrm {ZnO}$ in (**e**), R$_y^\mathrm {ZnO}$ in (**f**). (**c**) and (**g**) Extinction ratio of the reflected power between the two polarization, EXT  = 10log$_{10}(\mathrm {R}_x/\mathrm {R}_y)$ saturated in [0–15] dB for the TiO_2_ and ZnO cases with variable cavity thickness in the range $H=135{-}550$ nm, EXT$^\mathrm {TiO_{2}}$ in (**c**) and EXT$^\mathrm {ZnO}$ in (**g**). (**d**) and (**h**) Wavelength dependence of the extinction ratio in the range $\lambda$ = [1–4] μm for the thinner TiO_2_ (**d**) and the thicker ZnO (**h**) case calculated at an optimum cavity thickness *H*.

More details regarding the impact of the insulating cavity thickness and the performance of the metasurface as a polarizer, as well as the impact of the deposition material, TiO_2_ or ZnO, are presented in Fig. 4. There, in Fig. 4a,b,e,f, we show the reflected power, *R*, with respected to the cavity thickness varying in the range $H$ = [135–550] nm for both polarizations, $R_x$ and $R_y$, in the wavelength range $\lambda$ = [1–4] μm. In particular we plot the normalized reflected power for two plasmonic gap metasurfaces, of variable TiO_2_ (top panels, i.e., Fig. 4a,b) and ZnO (bottom panels, i.e., Fig. 4e,f) insulating cavity thickness in the range $H=[135{-}550]$ nm for both polarizations. We initially observe that since ZnO is a less optically dense material than TiO_2_ ( $n_{\mathrm {TiO_{2}}}$ = 2.7, $n_{\mathrm {ZnO}}$ = 1.95), the resonances of the former shift to lower wavelengths than the latter. This is to be expected for a Fabry-Perot type cavity whose resonances are, in quality, found at wavelength positions obeying the formula: $w 2\pi / \lambda n_{GSP} = m \pi -\phi$, where *w* is the feature width, *m* the integer order, $n_{GSP}$ is the effective gap mode index which is proportional to the index of the infiltrating material and $\phi$ is the overall phase shift at the boundaries^44^. Looking at the reflection of the TiO_2_ cavity (top panels) we observe that for the normal to the nanowires polarization (R$_x^\mathrm {TiO_{2}}$ in Fig. 4a), in thinner samples, i.e., when the insulating cavity thickness is approximately in the range $H=$ [135–200] nm, we obtain the prevalence of the magnetic dipole-type mode that forces zero reflection, while for thicker samples Fabry-Perot resonances take action and magnetic dipole-type mode appears as a sharp Fano-type resonant feature. On the other hand, we see that for the parallel to the nanowires polarization, the reflected power, R$_y^\mathrm {TiO_{2}}$ in Fig. 4b, presents generally broader and robust reflection dips (zeros) coming from Fabry-Perot resonances. The performance of the structures as polarizers for both insulating cavity cases TiO_2_ and ZnO is shown in Fig. 4c,g, respectively. There we plot the extinction ratio of the reflected power between the normal and parallel polarization, EXT = $|10\log _{10}(\mathrm {R}_x/\mathrm {R}_y)|$ with respected to the insulating cavity size in the wavelength range $\lambda$ = [1–4] μm, saturated in the range [0–15] dB, for TiO_2_ and ZnO cavity (EXT$^\mathrm {TiO_{2}}$ in Fig. 4c and EXT$^\mathrm {ZnO}$ in Fig. 4g). As observed in Fig. 4, the structures present several opportunities for polarizing applications that come from the resonant, polarization-sensitive absorption. For thinner samples, the normal to the nanowires polarization is absorbed with a narrowband peak, while the reflected power of the parallel polarization is over 50% (see Fig. 4a for the case of TiO_2_). For example, as seen in Fig. 4d, for the TiO_2_ case and cavity thickness $H$ = 165 nm the metasurface presents a 40 dB maximum extinction ratio at $\lambda$ = 1.410 μm and a 10 dB bandwidth equal to 30 nm, rendering the structure ideal for polarizing narrowband filters. The reflection levels of the parallel polarization are in the order of 60%. The thinner samples have relevant small tolerance to dielectric cavity size variation mainly due to the fact that the parallel polarization enters a resonance at these wavelengths and thus the reflected power decreases. On the other hand, for the thicker samples, the performance seems to be more robust. For example as seen in Fig. 4h, for the ZnO case and cavity thickness $H$ = 520 nm, the metasurface presents a 40 dB maximum extinction ratio at around $\lambda$ = 2.4 μm with a 10 dB bandwidth equal to 300 nm, while the reflection levels of the normal polarization remain high, greater than 90%, within a more extended gap range, rendering the structure ideal for polarizing filters. Based on the above analysis we selected to fabricate two samples, one thinner with TiO_2_ insulating cavity, aiming absorption of the polarization normal to the nanowires, **E** = $E_x$, and thickness $H$ = 165 nm, and one thicker of ZnO insulating cavity, aiming absorption of the polarization parallel to the nanowires, **E** = $E_y$, and thickness $H$ = 520 nm.

## Sample characterization

**Figure 5:**
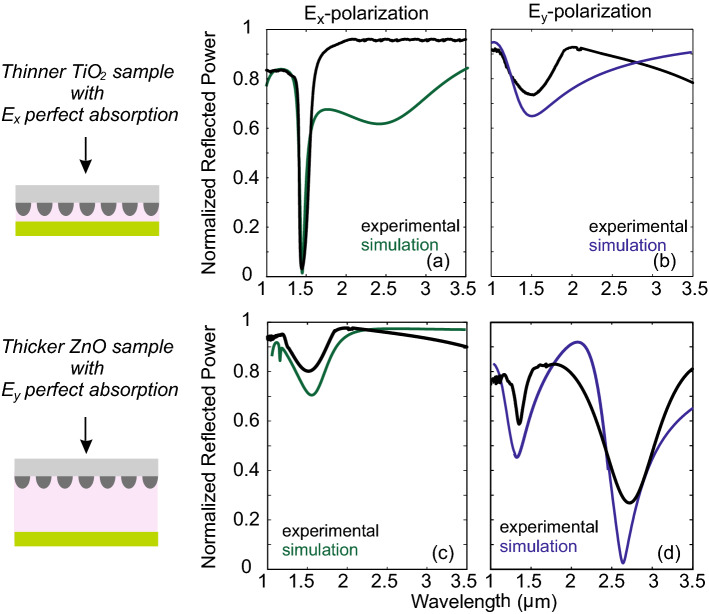
FTIR experimental measurement of the reflected power (black lines) for the two fabricated samples, the thinner TiO_2_ cavity sample (top panels) and the thicker ZnO cavity sample (bottom panels) for the two polarizations, **E** = $E_x$ [(**a**) and (**c**)] and **E** = $E_y$ [(**b**) and (**d**)]. The measurements are corroborated by the corresponding simulation (green and blue curves).

The samples were fabricated following the procedure described above and characterized through Fourier-transform infrared spectroscopy (FT-IR). The characterization measurements were performed using a Bruker Vertex 70v FT-IR vacuum spectrometer attached to a Bruker Hyperion 2000 infrared microscope, using a broadband KBr, an air cooled tungsten infrared source and a LN2 cooled Mercury-Cadmium-Telluride (MCT) detector and a set of two Specac KRS-5 wire grid polarizers. In each measurement, interferograms were collected at 4 cm^−1^ resolution (32 scans), apodized with a Blackman-Harris function, and Fourier-transformed with two levels of zero filling to yield spectra encoded at 2 cm$^{-1}$ intervals. Before scanning the samples, a background measurement was recorded using a Thorlabs’ Mid-Infrared Enhanced Gold Mirror (average reflectance > 98%), and each sample spectrum was obtained by automatic subtraction of it. The measurements of the normalized reflection in the wavelength range $\lambda$ = [1–3.5] μm for the thinner sample of TiO_2_ insulating cavity is presented in Fig. 5a,b (black curves) for the normal and the parallel to the nanowires polarization, respectively. The corresponding results for the thicker sample of ZnO_2_ insulating cavity are shown in Fig. 5c,d. The results stand in good agreement with the simulated response particularly regarding the position of the resonances, also provided in Fig. 5. Deviations regarding the amplitude may be attributed to various parameters such as a nonuniform cavity, a nonuniform back-reflector, nonuniform shape and size of the nanowires, etc. For the fitting of the experimental data we used $h=100$ nm, $w=140$ nm and periodicity of 520 nm (see Fig. 1d for the definition of *w*, *h*, *a*) while in the thinner TiO_2_ cavity sample the fitting height is equal to $H=175$ nm and in the thicker ZnO cavity sample the fitting height is equal to $H=530$ nm, allowing a deviation of 5%.

As expected by the theoretical investigation, in the thinner sample we experimentally observe a sharp, narrowband, reflection dip in the normal to the nanowires polarization, **E** = $E_x$, around 1450 nm, as a result of near unity absorption, while the the normal polarization reflection is as high as 70%, translated in a narrowband extinction ratio of 12 dB. In the thicker sample, the parallel to the nanowires polarization, **E** = $E_y$, presents two Fabry-Perot resonances as predicted by the theory, with a wavelength shift compared to the thinner case, one around 2600 nm and one around 1450 nm. As seen in the experimental case, we did not achieve perfect absorption which may be connected to a variety of reasons. Generally, we anticipate discrepancies between the experiment and simulation since: (i) The nanowires are not absolutely uniform. As it is observed in Fig. 2b the nanowires are not absolutely uniform, with the filling factor and periodicity exhibiting considerable deviations. (ii) Due to the non-planar surface where sol-gel/spin-coating and spray pyrolysis takes place, variations in the thickness of the deposited layer are expected. This means that at the insulating layer and the back-reflector are not absolutely planar; a small degree of rippling following the valleys and peaks of the nanowires may be expected which should affect more the thicker sample. This degrades the experimentally measured extinction ratio of the structures which is measured equal to 6 dB. The small electromagnetic signature of the magnetic mode, point 2 in Fig. 3c, expected around 1200 nm is vaguely, if not, spotted in the measurements, possibly due to experimental infrastructure resolution constrains. However, this sample highlights the shift of the resonance with regards to the spacer thickness and the cavity infiltrating material properties. In all, it is experimentally verified that the LIPSS fabricated metasurfaces provide very well-defined resonances with controllable response, enabled by the variation of the thickness the insulating cavity, rendering the method suitable for the further production of metasurface related applications.

## Conclusions

In conclusion, we have presented an alternative, versatile, approach of fabricating gap surface plasmon metasurfaces. Our method is based on direct material processing using pulsed laser light. With this approach we developed metal-insulator-metal type plasmonic metasurfaces with a corrugated top layer of sinusoidal shaped periodic thin metallic wires. In particular we imprinted laser induced periodic surface structures in a top layer of 120 nm Ni and subsequently, we created an insulating cavity via ZnO or TiO_2_ deposition followed by Au evaporation. The metasurfaces provide polarization control enabled by the polarization sensitive resonant gap surface plasmons that lead to controllable perfect absorption. We fabricated samples of different cavity size exhibiting perfect or enhanced absorption of either the normal or the parallel to the nanowires polarization with a resonance shift mediated by the insulating cavity thickness. Fourier-transform infrared spectroscopy characterization measurements corroborated with a numerical analysis demonstrated that the approach results in the development of metasurfaces with very well-defined resonances and efficient polarization control in the near-IR and mid-IR spectra. With proper adjustments, the approach could be used for the implementation of metasurfaces with further functionalities.

## Data Availability

The datasets used and/or analysed during the current study available from the corresponding author on reasonable request
